# Obesity as a premature aging phenotype — implications for sarcopenic obesity

**DOI:** 10.1007/s11357-022-00567-7

**Published:** 2022-04-26

**Authors:** Emily Nunan, Carson L. Wright, Oluwayemisi A. Semola, Madhan Subramanian, Priya Balasubramanian, Pamela C. Lovern, Ibra S. Fancher, Joshua T. Butcher

**Affiliations:** 1 College of Veterinary Medicine, Oklahoma State University, Stillwater, OK USA; 2 Department of Physiological Sciences, Oklahoma State University, Stillwater, OK USA; 3 Department of Biochemistry and Molecular Biology, University of Oklahoma Health Sciences Center, Oklahoma City, OK USA; 4 Oklahoma Center for Geroscience and Healthy Brain Aging, University of Oklahoma Health Sciences Center, Oklahoma City, OK USA; 5 Department of Kinesiology and Applied Physiology, College of Health Sciences, University of Delaware, Newark, DE USA

**Keywords:** Obesity, Aging, Skeletal muscle, Diabetes, Oxidant stress, Nitric oxide, Inflammation, Sarcopenic obesity

## Abstract

Obesity and aging have both seen dramatic increases in prevalence throughout society. This review seeks to highlight common pathologies that present with obesity, along with the underlying risk factors, that have remarkable similarity to what is observed in the aged. These include skeletal muscle dysfunction (loss of quantity and quality), significant increases in adiposity, systemic alterations to autonomic dysfunction, reduction in nitric oxide bioavailability, increases in oxidant stress and inflammation, dysregulation of glucose homeostasis, and mitochondrial dysfunction. This review is organized by the aforementioned indices and succinctly highlights literature that demonstrates similarities between the aged and obese phenotypes in both human and animal models. As aging is an inevitability and obesity prevalence is unlikely to significantly decrease in the near future, these two phenotypes will ultimately combine as a multidimensional syndrome (a pathology termed sarcopenic obesity). Whether the pre-mature aging indices accompanying obesity are additive or synergistic upon entering aging is not yet well defined, but the goal of this review is to illustrate the potential consequences of a double aged phenotype in sarcopenic obesity. Clinically, the modifiable risk factors could be targeted specifically in obesity to allow for increased health span in the aged and sarcopenic obese populations.

## Introduction

**Fig. 1:**
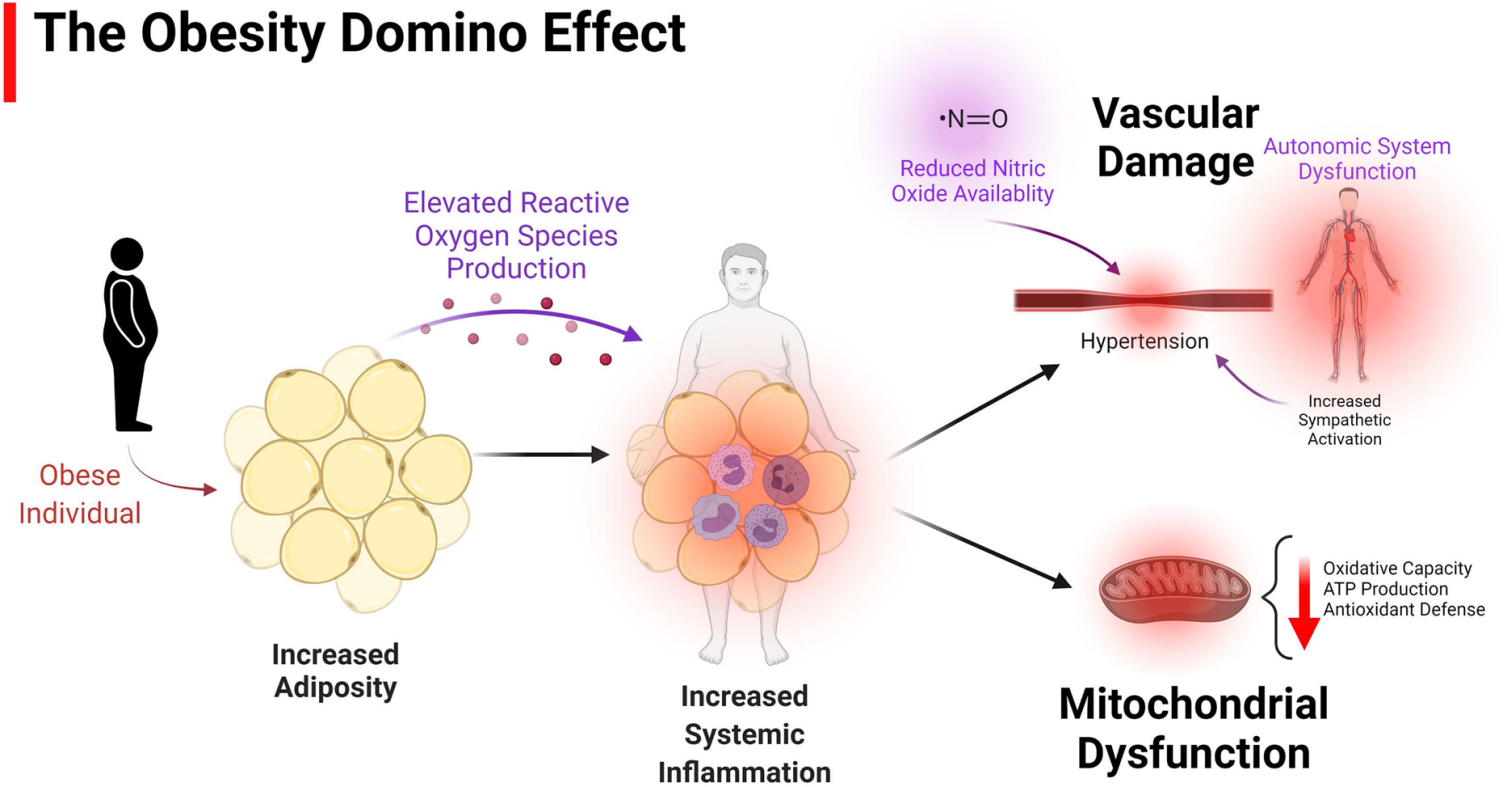
Schematically highlights common indices (starting with increased adiposity) that are altered in the aged and the obese. It highlights a potential domino effect of adiposity, which in both phenotypes subsequently are driven by increased ROS and systemic inflammation, ultimately contributing to vascular damage and mitochondrial dysfunction

In recent decades, two key pathologies have insidiously crept to the forefront of U.S. civilization: obesity and aging. Obesity is defined as a body mass index (BMI) of over 30, while the definition of overweight is a BMI of 25–30. Together, approximately 70% of U.S. adults are classified as overweight or obese and approximately 19% of children (age 2–19) are obese [1]. Obesity has largely been driven by imbalance between metabolic substrate consumption and utilization, namely the accessibility and affordability of a diet high in carbohydrates and fats, in combination with an increasingly sedentary lifestyle. The U.S. population is aging, and by 2030 the aged population (≥ 65) is expected to surpass the population of children for the first time in U.S. history. The prevalence of obesity has moved upwards in lockstep with population aging, with 35% of adults ≥ 65 also considered obese [2]. Growth of the aged population has been driven by remarkable medical advances that have slowed the progression of diseases that once significantly shortened lifespan (ex. cancer, cardiovascular disease). However, if one were to compare indices of aging against common risk factors accompanying obesity, one would observe that many of the key indices that define the pathology of aging also define the pathology of obesity (summarized in Fig. 1). The convergence of aging and obesity results in a unique pathology termed sarcopenic obesity, in which the complications of both states synergistically combine to cause loss of muscle and strength, increases in adiposity, and an inability to exercise [3]. Unfortunately, sarcopenic obesity is challenging to describe as a distinct pathology; it is a multidimensional syndrome that is loosely described as a state in which a patient presents simultaneously with muscle weakness and increases in adiposity. Thus, the conjunction of two diseases (obesity and sarcopenia) termed sarcopenic obesity has created a demanding field that merits further exploration and discussion. This review seeks to highlight several of those pathological similarities, in both human and animal models, and this is illustrated in Fig. 1. While it is currently under debate whether aging is preventable/reversible, obesity and many of its accompanied consequences (highlighted below) are measurable and are associated with modifiable risk factors. It is the intention of the following work to assist in identifying how obesity-focused cardiovascular treatment and research can better contribute to increasing not just lifespan, but healthspan in the aged population.

## Loss of skeletal muscle mass and function

Loss of skeletal muscle accelerates after the age of 50 (termed sarcopenia) and in sedentary individuals is responsible for a decrease of 1% of muscle mass per year. While the mechanisms underlying sarcopenia are still being delineated, research by Goodpaster et al. found aging adults lose muscle strength at a faster rate than their concomitant loss of lean muscle mass [4]. The significance of this finding demonstrates that aging adults’ muscle quality deteriorates in combination with loss of muscle quantity, accelerating the overall decline in muscle function. In aged muscle, an underlying factor contributing to sarcopenia is the decreased regenerative capability of muscle stem cells (termed satellite cells) [5, 6]. Although controversial, many researchers consider satellite cells to be an important component when studying sarcopenia. Satellite cells are necessary due to the dynamic and constant load placed upon skeletal muscle; thus, reduced regenerative capability of skeletal muscle will inevitably directly impair an individual’s muscle function and total mass. In both aging humans and rodent models, satellite cells are reduced in overall content and proliferative function, resulting in the loss of skeletal mass and function as observed with sarcopenia [6–11]. Much of the controversy surrounding satellite cells is due to the unknown significance of heterogeneity in the cell population, relative role of intrinsic versus extrinsic factors, and specific interactions with neighboring cells [6, 12]. A major factor contributing to the lack of satellite cell knowledge, especially in humans, is the difficulty of collecting samples. While this topic can be better defined in animals, advancements in muscle satellite cell research will enable us to fill the gaps in our knowledge and potentially alter the controversy surrounding them when considering sarcopenia in the human population.

Obesity is also accompanied by reductions in muscle mass and function [13, 14]. Research has shown that obese individuals are able to produce a greater force from a single contraction of anti-gravity muscle group when compared to a lean control; however when normalized to body mass, the force per kilogram body weight is reduced [15–17]. A greatly increased rate of fatigue in obese individuals has also been noted [16]. Muscle quality is diminished due to the negative metabolic and cellular changes of skeletal muscle, of which a significant portion is likely derived from the increase in nearby adipose tissue [18, 19]. Muscles not needed for weight bearing experienced a greater rate of impairment, since they are not as frequently utilized and typically not equipped to function optimally with increased adiposity. It should be noted that obesity also reduces satellite cell number and proliferative capacity, and slows their activation, in several rodent models of obesity (both diet-induced and genetic) — although evidence points toward the regenerative capability being preserved in these younger animals with exercise/loading [20–22]. Furthermore, it is well established in obese diabetic patients that satellite cells maintain an altered phenotype, even when removed from the environment, with sustained increases in inflammation and insulin resistance. While overall satellite cell content and function remains difficult to ascertain in humans, it has been found that the obese secretome significantly impairs myogenesis, but this effect is limited to old myoblasts compared to young myoblasts [23–26]. In summary, both aging and obesity result in not only a decrease of muscle mass, but also a reduction in muscle quality, and it is suspected that (largely based on animal studies) these deficits are driven by alterations to satellite cell content and function.

## Increased BMI

Body mass index is a focus of some debate as to whether it is an appropriate metric for overall assessment of healthy body composition. However, in the aged, BMI does begin to rise due to sustained adipocyte growth despite decreases in skeletal muscle. Evidence points toward decreased sex hormone production in aging as a contributor to the loss of muscle mass and accumulation of adiposity, although the specific hormones are distinct between men (low testosterone) and women (loss of estrogens) [27–29]. In the aging male, lower testosterone (hypogonadism) is a natural consequence of diminished testicular production and, as an anabolic steroid, subsequently results in lower muscle size and strength, impaired glucose homeostasis, and accelerated fat deposition [30, 31]. In the aging female, estrogens begin to decline at the onset of menopause and body composition subsequently shifts upwards in both overall mass and visceral adiposity, with evidence pointing toward alterations in estrogen receptor alpha (ERα) mediating these metabolic changes [32–34]. In obese individuals, BMI rises as a direct result of increased adiposity. Similar to the aged male, testosterone is lowered in the obese male, likely as a result of increased leptin and aromatase [35, 36]. Although typically obesity presents with increased estrogen, evidence also demonstrates decreases to the ERα receptor in obese women, mimicking the aging phenotype [37, 38]. Additionally, most obese individuals are less active, resulting in loss of muscle mass as fat cells increase in number and size. Obese individuals are also at a higher risk of other pathologies and have an increased chance of mortality when compared to lean individuals [39]. A similar overall pattern of decreased skeletal muscle mass combined with increasing adiposity is also observed in multiple rodent models, either genetic or diet-induced obesity (DIO) [13, 40]. As such, both aging and obesity have fundamentally similar effects in increasing BMI due to an overall increase in adiposity paired with a loss of muscle mass relative to overall weight.

## Adiposity distribution: subcutaneous versus visceral

To add to the complexity of sarcopenic obesity, there are considerable differences between where adipose tissue accumulates and its effects on overall homeostasis. Total body fat distribution can be further divided into subcutaneous adipose tissue (SAT) and visceral adipose tissue (VAT). SAT is adipose tissue deposited under the skin while VAT surrounds internal organs, especially the mesentery and omentum. Pathologically, VAT is deleterious as it fosters an inflammatory state, but SAT appears to be protective and, at least from an adiposity perspective, the “lesser evil” of the two [41]. The deposition of fat in the body is controlled by a multitude of factors, including the individual’s sex, age, nutrition, and metabolism. As an individual ages, VAT accumulation increases in humans [42, 43]. When analyzing the influence of age and sex on waist circumference, total body fat, VAT, and SAT, Kuk et al. found that aged men had a significant increase of VAT percentage when compared to young males and females, a result consistently observed across multiple studies [43]. While women did have a higher total body fat percentage when compared to men, their increase in VAT percentage was predominantly influenced by menopause instead of age. This study highlights how age (and sex) plays a considerable role in VAT accumulation. The importance of fat distribution is a rising area of concern in the scientific community focused on osteosarcopenic obesity, where deterioration of bone is included with the loss of muscle. The increase of abdominal and/or total body fat causes a rise in pro-inflammatory cytokines and hormone disruption, both of which are instrumental in driving osteosarcopenic obesity [44]. A key study by Perna and colleagues explored the differences between osteosarcopenic visceral obesity (OSVAT) and osteosarcopenic subcutaneous obesity (OSSAT) in humans [45]. Their results highlight a significant inflammatory phenotype with OSVAT, which was also accompanied with a higher risk of fractures and a worse metabolic profile when compared to the OSSAT phenotype. This data is also congruent with the concept of subcutaneous adiposity serving a “protective” role [46]. In a 2017 cross-sectional study performed with hospitalized Italian elderly patients, those with just sarcopenia were more vulnerable to fractures, edema, and inflammation than those with sarcopenic obesity, presenting an “obesity paradox.” Additionally, other studies have also indicated that higher SAT ratio does not lead to any significant pathology, especially when compared to those with a high VAT ratio [41, 45, 46]. The “obesity paradox” clearly demonstrates the need to address the current knowledge gap regarding the impact of total body fat distribution, sex, age, length of time of being obese, medications, and other factors that contribute to both lifespan and healthspan in sarcopenic obese patients [47]. An arguably important area of future risk assessment for those with sarcopenic obesity (or even just obesity) should be determining their VAT/SAT ratio, which can be performed via DXA, CT scan, trunk circumference, or an MRI scan [48, 49]. With the relatively recent discovery of how fat distribution is an important factor driving pathology, there is a great need for studies that closely examine the role of SAT and VAT in sarcopenic obesity.

## Alterations to autonomic function

Normal autonomic regulation is a critical determinant of cardiovascular health, involving the sympathetic and parasympathetic nervous system and comprising multiple spatially distinct receptors (nine receptors comprising α_1a,b,d_, α_2a–c_, and β_1–3_) that are targeted by catecholamines. These summate to impact overall blood pressure via peripheral resistance and cardiac function. One of the major clinical consequences of autonomic dysregulation, shared by both aging and obesity, is the development of hypertension. The aged are commonly afflicted with isolated systolic hypertension, of which a significant risk factor is sympathetic over-activation [50, 51]. Several lines of evidence ranging from direct nerve recordings to plasma norepinephrine (NE) levels support the key role of an overactive sympathetic nervous system in both aging and obesity. While there are regional differences in sympathetic outflow to different vascular beds, elevated sympathetic nerve activity (SNA) to the muscle is common to both pathologies [52–55]. In addition, chronic increases in SNA to the muscle cause internalization of the β-adrenergic receptors and decreased expression at the plasma membrane with age, termed “β-adrenergic desensitization” [51]. This results in unfavorable activation of α-adrenergic receptors, further augmenting the effects of SNA on vasoconstriction and in turn blood pressure [51]. Along with the dysregulation of receptors, clearance of plasma NE decreases significantly in the aged, causing prolonged activation of the cardiovascular system [56, 57]. The neural mechanisms underlying aging and obesity-induced increases in SNA are reviewed elsewhere [50, 51]. Obesity also results in an increased stimulation of the sympathetic system by adrenergic manipulation, commonly seen as hypertension [58]. Independent of direct effects on blood pressure, normotensive obese individuals still had a significantly increased muscle sympathetic nerve activity (measured by microneurography) when compared to lean normotensive individuals, suggesting that the underlying autonomic dysfunction is still present [59]. Contrary to the sympathetic system, interventions that activate the parasympathetic system including vagal nerve stimulation are shown to exert beneficial effects on the heart and vasculature in both obesity and aging [60–62]. The dysregulation of autonomic function noted in both aging and obesity contributes to unfavorable hypertension and an increased risk of cardiovascular disease, which does serve as the leading cause of preventable deaths globally [63].

## Reduced nitric oxide bioavailability

As discussed above, adrenergic dysfunction skews vascular function toward a chronic constricted state in aged individuals. This effect is compounded by a reduction in the bioavailability of nitric oxide (NO), a potent vasodilator [64]. Functionally, this reduction begins with decreases in the expression and activity of endothelial nitric oxide synthase (eNOS). Additionally, uncoupling of eNOS can reduce NO production, as it is one of the enzymes known to need multiple cofactors, making it susceptible to uncoupling when any of those are limited [65–69]. Beyond aged individuals having dampened NO anabolism, there is also an enhanced NO breakdown (linked to an increase of reactive oxygen species) [64]. For obese individuals, there is an obvious change in the cellular environment, producing conditions not suitable for optimal vascular function [70]. Similarly to aged individuals, obese individuals have an impaired NO production and utilization, much of which is secondary to increased ROS [71]. The NADPH oxidase family (NOX, isoforms NOX1–5) seems to be key as it is upregulated in obesity and is responsible for producing reactive oxygen species (superoxide and hydrogen peroxide). While the isoforms are not conserved across species (NOX5 is not found in mice or rats) and likely play organ-specific roles, NOX1, 2, and 5 are significant producers of superoxide with NOX4 predominantly producing hydrogen peroxide. In obese humans and rodents, targeting the NOX family reduces vascular dysfunction significantly [72, 73]. While the direct mechanism whereby obesity upregulates the NOX family remains elusive, its expression correlates very well with adiposity and glucose, both key indices that have observed increases in aging and obesity [74–76]. Thus, the decrease in NO bioavailability observed in obesity likely results from scavenging by ROS, as opposed to the altered production as seen in aging (although eNOS uncoupling can also occur in obesity) [77–79]. Beyond the periphery, microvascular endothelial dysfunction due to decreased NO bioavailability also affects brain perfusion and in turn leads to cognitive dysfunction in obesity. Tucsek et al. showed that aging further exacerbates obesity-induced decrease in NO bioavailability as reflected by impaired neurovascular coupling responses (a mechanism of moment-moment adjustment of blood flow to meet the nutritional demands of active brain regions) [80]. Chronic vascular dysfunction paired with cellular senescence in the brain has been noted in obese mouse models, causing obesity-induced sympathoexcitation [81]. Furthermore, hypertension induced by angiotensin II infusion (well characterized to be potentiated by NOX1) in an aged Alzheimer’s mouse model clearly showed increased cerebral microhemorrhages and was accompanied with functional deficits in movement [82]. Combined, these mechanisms suggest a positive feedback loop, where increases in adiposity drive microvascular dysfunction through reduced dilatory capacity, altering brain blood flow/function and amplifying the sympathetic tone to peripheral vasculature, leading to an overall increase in vascular resistance. When considering both obese and aged pathologies together, there is an additive effect of vascular impairment that will likely result in a greater risk of cerebral vascular disease and cognitive dysfunction in the sarcopenic obese population [39].

## Increased oxidative stress and inflammation

Oxidative stress refers to excess levels of reactive oxygen species, some of which are free radicals. Oxidative stress is the foundation of many pathologies observed in obesity and aging, through its effects to induce vascular dysfunction, disrupt biochemical processes, promote chronic inflammation, and impair mitochondrial function (to name a few) [83, 84]. As animals age, the increased incidence of biochemical mishap is a natural consequence of cellular replication [85]. Aging individuals experience an increase of reactive oxygen species production, which shift the body’s cellular processes toward a toxic environment. Some of the most notable alterations that occur with oxidative stress include apoptosis, necrosis, autophagy, and cellular senescence. Upon entering cellular senescence, cells change to a senescence-associated secretory phenotype (termed SASP), which alters their secretome profile to be much more inflammatory and promote an environment poised for tissue dysfunction. Cellular senescence is associated with the development of multiple age-related diseases and an upregulation of the DNA damage-response system causing inflammation [86]. Within aging, inflammatory cytokines are upregulated, including tumor necrosis factor-α (TNF-α) and interleukin-6 (IL-6) [87, 88]. IL-6 regulates the inflammatory C-reactive protein and promotes a chronic inflammatory response in humans [89]. Adiposity itself is an active endocrine organ, secreting adipokines that result in an unnatural environment promoting free radicals and inflammation. When measuring serum malondialdehyde (MDA), a biomarker of oxidative stress, obese humans have a statistically significant increase of MDA compared to lower BMI individuals [90]. Increased free radical production and inflammation are interrelated, and unfortunately, adiposity stimulates both detrimental factors. Oxidation of lipoproteins induces monocytes to release pro-inflammatory cytokines [91]. Along with the oxidized lipoprotein, adipose tissue itself secretes the inflammatory cytokines IL-6 and TNF-α, resulting in elevated levels in the obese [92]. Systemic and sustained inflammation and reactive oxygen species are detrimental to the health of any animal and the increased production in the obese and aged, in combination with downregulation of antioxidant defenses, is a pointed indicator of increased morbidity and mortality.

## Loss of glucose homeostasis

Maintenance of blood glucose homeostasis by insulin and glucagon is arguably one of the most important physiological processes in mammals. Glucose is the body’s main source of energy and is especially critical for brain function as the brain has little (if any) ability to synthesize it. Insulin, a hormone produced by the pancreatic β-cells, enables glucose absorption from the bloodstream by insulin-sensitive tissues such as muscle and adipose. Decreased sensitivity of insulin receptors results in chronic type 2 diabetes mellitus, characterized by excessively high blood glucose levels (hyperglycemia). Both obesity and aging are leading risk factors for type 2 diabetes. Indeed, data from the 2013–2016 NHANES show approximately 27% of US aged (> 65 years old) adults have diabetes [93]. The increased cellular senescence in the aged is thought to hinder pancreatic β-cell function [94]. In a mouse model with increased β-cell senescence by genetic manipulation, glucose homeostasis returned to normal levels after senolysis (clearance of the senescent cells) [94]. In a human study, insulin binding in peripheral tissue was decreased in aged participants, demonstrating an age-related decrease in insulin sensitivity [95]. Thus, age-related loss of glucose homeostasis can be attributed to β-cell dysfunction coupled with lowered insulin sensitivity. While not all obese individuals are diabetic, obesity is an independent risk factor for diabetes. Indeed, within the type 2 diabetic population, a high percentage (52%) meet the definition for obesity (BMI > 30); when criteria are broadened to include overweight individuals (BMI > 25), 90% of type 2 diabetic patients are included [96, 97]. Of great concern is that increasing BMI, especially at younger ages, will dramatically increase lifetime risk for diabetes [98]. Increased adipose tissue is an important causal factor for glucose dysregulation. Adipocytes of obese individuals store large amounts of lipoproteins and have a limited ability to absorb postprandial increases in circulating lipids. This leads to fat deposition in non-adipose tissues such as skeletal muscle, which is directly linked to insulin resistance in those tissues [99]. Lastly, increased inflammation, which is noted in both aged and obese individuals, disrupts cellular insulin sensitivity and contributes to insulin resistance. In a diabetic obese mouse model, treatment with an anti-inflammatory drug notably decreased insulin resistance, due to improved cell signaling [100]. In summary, many of the factors that contribute to and drive the progression of impaired glucose homeostasis are similar in both aging and obesity.

## Loss of mitochondrial function

Overall, mitochondrial dysfunction in the aged is likely a consequence of the integration of many of the abovementioned pathologies. Originally, it was theorized that loss of mitochondrial function correlated with increased inflammation and oxidative stress, but more recent studies have favored the theory of age-related dysfunction as a combination of the aforementioned pathologies and the rise of mitochondrial DNA (mtDNA) dysfunction [101, 102]. It is well established that mitochondrial content in skeletal muscle decreases with aging [103, 104]. Short et al. observed that, compared to young healthy counterparts, even healthy aged patients had decreased skeletal muscle mitochondrial function that correlated well with age-related decreases in mtDNA, mitochondrial proteins, mRNA, and mitochondrial ATP production. These conditions occurred in combination with a rise in oxidative DNA damage, as assessed by 8-oxo-deoxyguanosine [102]. Additional studies have examined if intramyocellular lipids (IMCL) in the ultrastructure of skeletal muscle could impact mitochondrial function in aging, as metabolic syndrome increases IMCL [105]. Crane et al. found that, while aging reduces mitochondrial content in the aged population, the IMCL droplets are also significantly larger. These changes are directly associated with changes in mitochondrial dynamics and reduced mitochondrial ATP production in aged individuals [105]. However, in one study, age-matched elderly pre-frail subjects had significantly decreased skeletal muscle mitochondrial function in vivo compared to active elderly controls, along with decreased mtDNA. These results demonstrate that exercise in the elderly can help counteract the effects of aging on mitochondria [106]. This is further supported by the work of Distefano et al., who showed that older active individuals had improved skeletal muscle performance compared to an older sedentary group, in addition to improved mitochondrial energetics and lower adiposity [107]. Together, this work suggests that mitochondrial dysfunction may be directly linked to physical (in)activity as opposed to an endogenous chronological aging process. Obese individuals have also been reported to have downregulation of mitochondrial oxidative capacity when compared to lean individuals [108]. In obesity, excess caloric intake and/or high-fat diets overwhelm mitochondrial and cellular metabolic processes with a plethora of substrates, leading to an overproduction of reactive oxygen species (ROS), which cause further mitochondrial damage and dysfunction [109]. Potes et al. specifically targeted an overweight and aged population to see if mitochondrial dynamics would be altered without the presence of true obesity. The study showed significant impairment in ATP production, a further reduction in pyruvate kinase activity, and accompanying protein oxidative damage compared to aged controls [110]. Interestingly, measures of mtDNA were increased with obesity but were also accompanied by a reduction of mitophagy markers. Taken together, this seems to suggest that obesity may upregulate mitochondrial biogenesis while impairing mitophagy, the long-term result of which would be accumulation of dysfunctional mitochondria with aging. While it is certainly interesting to speculate regarding whether mitochondrial (dys)function could be improved or prevented in aging (and exercise/activity does look promising) when taken together, the dysregulation of the “powerhouse of the cell” in both aging and obese individuals propagates underlying dysfunction, regardless of whether the cell type utilizes the mitochondrial output as a source of energy or a signaling molecule.

## Discussion and future impact of sarcopenic obesity

**Fig. 2:**
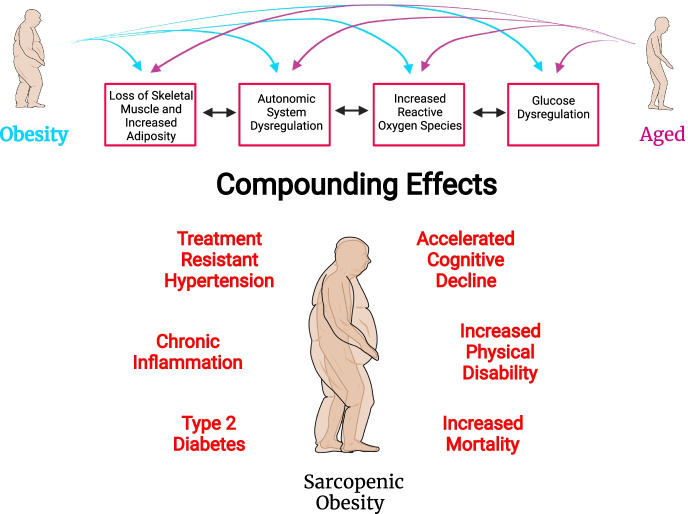
Highlights the “double aged” phenotype that could be hidden within sarcopenic obesity. Namely, the additive nature of skeletal muscle dysfunction, autonomic dysfunction, increased ROS, and glucose dysregulation will drive entrenched hypertension, chronic inflammation, diabetes, and increased disability, and accelerated cognitive decline, ultimately resulting in increased mortality

To conclude, much of the research into obesity and its accompanying cardiometabolic disease demonstrates very clear and important correlations to aging. However, the long-term consequences of exposing the human body and organ systems to lifelong obesity (or premature aging) and then subsequently entering an aging paradigm would likely lead to a deeply entrenched disease state that would be highly resistant to reversibility or rescue, either pharmacologically or with lifestyle changes. It is very clear that as the number of co-morbidities increase with sarcopenia, there is an increased risk of mortality [111]. Arguably, obesity is one of the most critical co-morbidities to be taken into account when analyzing sarcopenia in an individual. While there are certainly large gaps of knowledge regarding mechanistic links and crucial drivers of pathology between obesity and aging, we hope this review addressed similarities that could drive pathology in the sarcopenic obese population. There is also a considerable lack of studies investigating the effects of intentional weight loss, via exercise and/or nutrition, in individuals with sarcopenic obesity [112]. However, even with the minimal studies examining the effects of weight loss in sarcopenic obesity, there is evidence that directly targeting adiposity and skeletal muscle health (by either aerobic or weight training) can assist with maintaining glucose regulation, decrease the use of hypertension medication, and improve control of pulmonary function in obese or aged individuals [113–117]. Regardless, there is a growing population of patients presenting with sarcopenic obesity, a dual condition in which obesity and sarcopenia synergistically present with increased morbidity, disability, and mortality [3, 118, 119]. Indeed, end-organ damage within sarcopenic obesity has not been well characterized and thus may have progressed to a point of no return, regardless of treatment. The increase in visceral adiposity, dysfunction of skeletal muscle, autonomic signaling, vascular maintenance, and an inability to maintain glucose homeostasis all contribute to the severity of the pathologies (represented schematically in Fig. 2, where sarcopenic obesity has an “aging” phenotype hidden within obese physiology). As such, given the increasing and sustained rates of obesity in both adults and children, it is a medical necessity that we identify and target key outcomes (blood pressure, renal function, glucose homeostasis, vascular health, muscle mass) that must be preserved for overall healthspan and lifespan to be maintained in the aged.
